# An interview with Lucio G. Costa and Michael Aschner, section editors for toxicology

**DOI:** 10.1186/2050-6511-13-16

**Published:** 2012-11-15

**Authors:** Lucio G Costa, Michael Aschner

**Affiliations:** 1 Department of Environmental and Occupational Health Sciences University of Washington, 4225 Roosevelt #100, Seattle, WA, 98105, USA; 2 Vanderbilt University Medical Center, 2215-B Garland Avenue, 11415 MRB IV, Nashville, TN, 37232-0414, USA

## Abstract

Lucio G. Costa is currently Professor of Environmental and Occupational Health Sciences at the School of Public Health at the University of Washington. Dr. Costa is a renowned neurotoxicologist whose research interests are focused on understanding the role of neurotoxic substances in neurodevelopmental disorders and other neurological, neuropsychiatric and neurodegenerative diseases. Dr Costa’s research laboratory makes use of a variety of *in vivo* and *in vitro* cell culture systems, transgenic animal models and imaging techniques to study the cellular, biochemical and molecular mechanisms of neurotoxicity.

Michael Aschner is the Gray E. B. Stahlman Professor of Pediatrics and Pharmacology at Vanderbilt University School of Medicine as well as a Senior Investigator at the Kennedy Center for Research on Human Development**.** Dr Aschner’s research group has a particular interest in the neurobiology and physiology of astrocytes and the signaling mechanisms associated with central nervous system injury. Dr Aschner’s laboratory studies metal uptake and distribution in the brain, investigating the mechanisms of transport of methylmercury and manganese across the capillaries of the blood–brain barrier. His research utilizes various experimental models (C. elegans, tissue cultures and rodents) to understand the acute toxicity of manganese deposition in the brains of human neonates.

In this interview we find out a little more about the key issues in the field of toxicology research.

## How did you first become interested in pharmacology and toxicology?

Lucio: I first became interested in pharmacology, a discipline which I thought was at the crossroad of medicine, chemistry and biology. After my doctorate in pharmacology, I was asked to consider a postdoctoral position in a new laboratory at the University of Milano, which was attempting to “launch” research in toxicology. I found the field fascinating and well trenched into everyday life. Indeed my first project was related to a color used in a very common alcoholic drink.

Michael: As a graduate student in Neurobiology and Anatomy I rotated in the laboratory of the late Dr. Patricia Rodier. She was studying at the time the effects of methylmercury on neurodevelopment, addressing the antimitotic effects of this metal. I knew immediately this was an area of interest to me and have stuck with it since, through my postdoctoral years with “Mr. mercury”, Dr. Thomas Clarkson and independently thereafter.

## Why is it an exciting time to be involved in toxicology in particular?

More than other areas of research, toxicology is interdisciplinary. While solid foundations in chemistry and biochemistry, biology, genetics, pathology and pharmacology are essential, they need to be integrated by knowledge and expertise in diverse areas such as exposure assessment, epidemiology, and public health. In addition, there is a need to comprehend and integrate the new “omics” disciplines into toxicological issues. The ultimate goal is that of protecting the public from the adverse effects of chemicals, by applying sound scientific methods, and avoiding irrational and unjustified fears.

## What challenges and developments can we expect to see in the next few years in this field?

The main challenge probably remains that of determining to which extent and how exposure to chemicals may contribute to disease. Two areas are particularly challenging, related to early development and to aging. Exposure *in utero* or in early childhood to chemicals is strongly suspected to contribute to neurodevelopmental disorders, which appear to be increasing at an alarming rate, and to other disease of adulthood, particularly diabetes and obesity. With the longer life expectancy, we have also witnessed a substantial increase in neurodegenerative diseases. Whether some of these may be due to environmental exposures throughout life, and to gene-environment interactions, is an area of great importance. Additional challenges for toxicology are, for example, the need to develop new ways of testing for toxicity of chemicals which should be faster, predictive, and possibly inexpensive, and to respond to the increasing use of new materials, particularly the nanomaterials, which are finding an increasing number of applications.

## How can novel toxicology research contribute to meeting these challenges?

Toxicologists have come to realize that traditional toxicity testing, while it has served well the community over the years, is too cumbersome for the thousands of chemical entities on the market. Additionally, traditional toxicity testing is time-consuming, costly, and utilizes a large number of animals. One of the novel areas of research in toxicology is that of finding perturbation in certain cellular pathways which may serve of “fingerprints” of an ensuing disease. This task, which is rather easy to envision, is proving to be extremely challenging in its realization. Another area of research, which is not new but that have not lost its importance, is that of biomarkers, i.e. of early indicators of disease. Such measurements, as all other aspects of toxicology, would much benefit for increasing mechanistic research. As indicated, animal use is under scrutiny for a number of reasons, hence the challenge to developing and particularly, to validating new alternative methodologies which utilize *in vitro* systems or alternative animal species such as worms (*C. elegans*).

## How does toxicology contribute to the development and safety assessment of new therapeutic interventions?

Numerous companies are working to develop novel and innovative drugs for treating diabetes, hypertension, obesity, heart failure, hyperlipidemia, neurodegenerative diseases and transplant rejection, to name a few. Current approaches to toxicity testing and decision-making aim to identify and/or support development of the best lead candidates for human use. The identification of lead candidate and their therapeutic efficacy is largely based on animal studies, where toxicology plays a key role. The current approach to toxicity testing relies predominantly on studies that aim to evaluate observable outcomes in whole animals, such as clinical signs or pathologic changes that are indicative of a disease state. In general, failure in toxicology studies also represents the primary reasons for the attrition of the most promising therapeutic agents from the drug development pipeline. It is the lack of or the observation of treatment-related toxicity as evidenced most often in animal toxicology studies that determines whether a candidate drug will move along the pipeline to the next step, human trials.

## What does the future hold for toxicology research and what do you think are its limitations?

Toxicity testing of both potential therapeutics as well as environmental contaminants is presently at a crossroads. With the advents in biology and biotechnology, toxicogenomics, epigenetics, and computational toxicology, toxicity testing is poised to move from the traditional animal testing (*in vivo*) to *in vitro* systems. Ideally, the testing will likely take advantage of cell lines of human origin. While by inference this approach will curtail the usage of animals in toxicity testing, it has yet to be determined how the new in vitro testing will meet challenges in evaluating various life stages, numerous health outcomes, and large numbers of untested chemicals. The new assays may be suited to assess measure simple processes, such as binding of drugs or environmental agents with cellular proteins and changes in gene expression caused by that binding, or they may measure more integrated responses, such as cell division and cell differentiation. Nevertheless, in order to be validated it would have to be determined that they replicate in vivo toxicity, a difficult task, as the in vitro systems represent a highly reductionist approach. Notwithstanding this obstacle, toxicity tests are expected to use high-throughput methods, assessing cytotoxicity, cell proliferation and apoptosis. Over time, as these systems become refined, the need for traditional animal testing should be greatly reduced.

## Are there any particular papers you would like to see submitted to your section?

We are interested in original research and review papers that address the interactions of chemicals with biological systems. The manuscripts may include basic research of molecular mechanisms underlying normal and abnormal physiology to define novel therapeutic targets; translational research to adapt observations from the laboratory to patients; clinical research to examine the behavior of novel therapeutics in humans; basic research to identify molecular mechanisms and targets of xenobiotics; and population research to identify health outcomes associated with exposures to environmental agents and xenobiotics. Articles on promising new approaches in alternative toxicity testing are also welcome.

## Competing interests

Lucio G. Costa and Michael Aschner are Section Editors on *BMC Pharmacology and Toxicology*.

## Authors’ contributions

LGC and MA wrote and approved the final text.

